# Ubiquity of synonymity: almost all large binary trees are not uniquely identified by their spectra or their immanantal polynomials

**DOI:** 10.1186/1748-7188-7-14

**Published:** 2012-05-21

**Authors:** Frederick A Matsen, Steven N Evans

**Affiliations:** 1 Program in Computational Biology, Fred Hutchinson Cancer Research Center, Seattle, Washington, USA; 2 Department of Statistics, University of California at Berkeley, Berkeley, California, USA

## Abstract

**Background:**

There are several common ways to encode a tree as a matrix, such as the adjacency matrix, the Laplacian matrix (that is, the infinitesimal generator of the natural random walk), and the matrix of pairwise distances between leaves. Such representations involve a specific labeling of the vertices or at least the leaves, and so it is natural to attempt to identify trees by some feature of the associated matrices that is invariant under relabeling. An obvious candidate is the spectrum of eigenvalues (or, equivalently, the characteristic polynomial).

**Results:**

We show for any of these choices of matrix that the fraction of binary trees with a unique spectrum goes to zero as the number of leaves goes to infinity. We investigate the rate of convergence of the above fraction to zero using numerical methods. For the adjacency and Laplacian matrices, we show that the *a priori *more informative immanantal polynomials have no greater power to distinguish between trees.

**Conclusion:**

Our results show that a generic large binary tree is highly unlikely to be identified uniquely by common spectral invariants.

## Background

Tree shape theory furnishes numerical statistics about the structure of a tree [1,2]. (Because we are interested in applications of tree statistics to trees that describe the structure of branching events in evolutionary histories, we will, for convenience, always take the term *tree *without any qualifiers to mean a **rooted, binary tree without any branch length information or labeling of the vertices**.) Such statistics have two related uses. Firstly, they can be used in an attempt to tell whether two trees are actually the same and, secondly, they can be used to indicate the degree of similarity between two trees with respect to some criterion.

Examples of the latter use are the testing of hypotheses about macroevolutionary processes and the detection of bias in phylogenetic reconstruction. Historically, numerical statistics for such purposes have attempted to capture the notion of the *balance *of a tree, which is the degree to which daughter subtrees are the same size. The balance is typically measured by ad-hoc formulae that are often selected for statistical power to distinguish between two different distributions on trees [3,4]. In previous work we investigated the possibility of describing the shape of the tree using a list of numbers rather than just a single number [5,6].

A mathematically "canonical" approach to finding a list of such numbers is to use information derived from matrix representations of the trees. We first describe the matrix representations of a tree that we will consider.

In algebraic graph theory [7], the basic matrix associated to a graph is the *adjacency matrix A*(*G*), whose *ij*^th ^entry is one if *i *and *j *are connected by an edge, and zero otherwise. From a probabilistic point of view, the more natural matrix to associate with a graph is the *Laplacian matrix L*(*G*), which is the infinitesimal generator of the natural random walk on the graph and is given by *A*(*G*) - *D*(*G*), where *D*(*G*) is the diagonal matrix of vertex degrees. It is clear that a graph can be recovered from either its adjacency of Laplacian matrix. Some authors, such as [8], define the Laplacian to be *D*(*G*)^-1/2^*L*(*G*)*D*(*G*)^1/2^. Note that this difference is not relevant if one is only considering characteristics of the matrix *L *such as eigenvalues that are invariant under similarity transformations.

Readers in the phylogenetics community may be more familiar with the *pairwise distance matrix *[1,9]. The distance matrix *P *given a leaf-labeling 1, . . ., *n *has as its *ij*^th ^entry the length of the path between leaf *i *and leaf *j*. Any leaf-labeled tree is uniquely determined by its distance matrix. These matrices have also been extensively studied as discrete metric spaces [10,11].

The definition of the adjacency and Laplacian matrices requires a numbering of the vertices, while the definition of the distance matrix requires a numbering of the leaves. Because we are considering unlabeled trees (that is, we identify trees that are equivalent in the usual sense of graph-theoretic isomorphism), we are only interested in tree statistics that are invariant under renumbering. Algebraically, this means that we are only interested in features of the associated matrix that are unaffected by similarity transformations via a permutation matrix. The most obvious such statistics are the eigenvalues.

The adjacency and Laplacian matrices and their eigenvalues are familiar objects in the area of spectral graph theory [7,8,12]. The eigenvalues of the adjacency matrix tend to contain combinatorial information about the graph, such as bounds on the chromatic number. The eigenvalues of the Laplacian give information of a more geometric flavor, such as the equivalent of the surface area to volume ratio of subgraphs of a graph. As well as having connections to the theory of random walks on graphs, the Laplacian eigenvalues can be used to define the expander graphs, an important class of graphs that have applications in coding theory. Therefore, it would not be too surprising if the these eigenvalues were a convenient way to summarize information about a tree, thus giving a nice collection of tree statistics.

Similarly, it seems plausible that the eigenvalues of the pairwise distance matrix could contain quite a lot of information about the tree that could be used to compare trees. Moreover, although the distance matrix formally contains the same information as the adjacency or Laplacian matrices, the transformation that takes the distance matrix to one of the other two is distinctly non-linear, and hence there is no reason to believe that there is any simple connection between the corresponding eigenvalues.

We demonstrate below that not only do there exist pairs of trees that have the same spectrum as another tree for the adjacency, Laplacian, and distance matrices, but that this is the rule rather than the exception as the trees become large, in the sense that the fraction of trees with a given number of leaves that have a unique adjacency, Laplacian, or distance spectrum goes to zero as the number of leaves goes to infinity.

**Figure 1:**
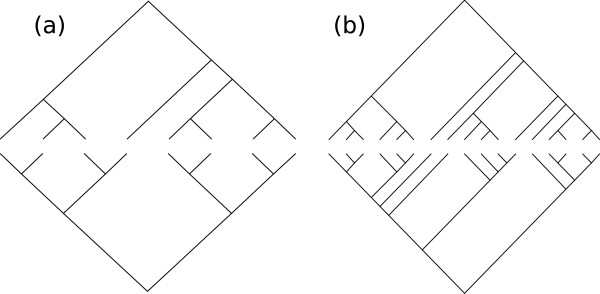
**Pairs of trees with similar algebraic properties. **Figure (a) shows the smallest pair of rooted binary trees with the same adjacency and Laplacian spectrum; these trees do not have the corresponding exchange property. Figure (b) shows two trees with the exchange property for the adjacency, Laplacian, and pairwise distance matrices.

The basic methodology that we use to prove this result was first established in [13] and developed in [14] for general (that is, not necessarily bifurcating) graph-theoretic trees in the case of the adjacency and Laplacian matrices. The present paper provides the first results of this type concerning rooted bifurcating trees, as well as the first examination of such results for the pairwise distance matrix. The key idea is to establish that certain pairs of trees *T*_1 _and *T*_2 _have the following *exchange property *for a given matrix representation: that exchanging *T*_1 _for *T*_2 _as subtrees of a given tree does not change the spectrum for that matrix representation. This is a stricter requirement than simply having the same spectrum (Figure 1). It then becomes a matter of showing that the number of trees with a given number of leaves is asymptotically of larger order than the number of trees with the same number of leaves that don't have a particular subtree. For this we build on the generating function argument used in [15] for asymptotic estimates of the number of unlabeled rooted bifurcating trees (see the section *Asymptotic numbers of trees*).

One possible explanation for this phenomenon is that two diagonalizable matrices have the same spectrum if they are similar via an arbitrary similarity transformation rather than just via a permutation transformation, and this suggests considering features of a matrix that are invariant under permutation similarities but not more general ones. We will now describe a feature of a matrix, its *immanantal polynomial, *that has this property.

The *immanant *is a generalization of the determinant. Recall that the determinant of a matrix *A *= (*a*_*ij*_) is given by

$$\text{det}\left(A\right):=\underset{\sigma \in S_{n}}{\sum }\text{sgn}\;\left(\sigma \right)\underset{i}{\prod }a_{i\sigma \left(i\right)},$$

where the sum is over the symmetric group of permutations of {1, 2,..., *n*} and sgn(*σ*) is the *sign *of the permutation *σ.*

The function sgn is a particular example of a *character *of an *irreducible representation *of the symmetric group. It would take us too far afield to define these notions here, but excellent treatments may be found in [16-19]. We note, however, that the irreducible characters are constant on the conjugacy classes of the symmetric group (recall that two permutations belong to the same conjugacy class if and only if they have the same cycle structure) and they form a basis for the vector space of functions with this property (the *class functions).*

Our use of characters is simply to define the immanant

$$I_{\chi }\left(A\right):=\underset{\sigma \in S_{n}}{\sum }\chi \left(\sigma \right)\underset{i}{\prod }a_{i\sigma \left(i\right)}$$

of a matrix *A *for the irreducible character χ. A discussion of immanants may be found in [20,21]. The *immanantal polynomial *for a character *χ *of a matrix is the corresponding generalization of the characteristic polynomial; that is, it is the polynomial $x\mapsto I_{\chi }\left(xI-A\right)$. Because the characters are class functions, an immanantal polynomial is invariant under similarity by permutation matrices, but it will not typically be invariant under more general similarities.

Unfortunately, as we show in Lemma 2, for either the adjacency or Laplacian matrix the following two conditions on a pair of trees are equivalent:

• the spectra are equal,

• the immanantal polynomials are equal for all irreducible characters.

Consequently, the immanantal polynomials for the adjacency and Laplacian matrices provide no more distinguishing power than the spectra and, in particular, a vanishing fraction of large trees have a unique set of immanantal polynomials for these matrices. We do not know if the same fact is true for the immanantal polynomials of the distance matrix.

Our main result is thus the following.

**Theorem 1**. *Let t*_*n *_*be the number of trees with n leaves. For either the adjacency, Laplacian, or pairwise distance matrix, let l*_*n *_*be the number of trees with n leaves that do not share their spectrum with another tree. Then, the fraction l*_*n*_*/t*_*n *_*goes to zero as n goes to infinity. For the adjacency and Laplacian matrices, the same result holds if we replace the spectrum by the complete set of immanantal polynomials*.

The rate of convergence of the fraction in Theorem 1 is also of interest. If it is extremely slow then the existence of trees with shared spectra may not be practically relevant for the construction of informative tree shape statistics. We investigate this matter numerically towards the end of the paper.

Several other research groups have investigated problems that are related to, but different from, those investigated here. Steyaert and Flajolet [22] investigate the occurrences of subtrees in the case of "planar" trees, i.e. trees that are equipped with an order of subtrees at every internal node. These planar trees are substantially easier to analyze: for example, there is a nice closed form generating function for the numbers of such trees (the Catalan numbers). In contrast, the generating function for the number of trees considered here is only given as the solution of a functional equation and there is no closed form expression for the numbers of such trees. Graham and Lovasz [23] investigate the spectra of distance matrices of trees, but their distance matrices are defined in terms of vertex-to-vertex distances, rather than the leaf-to-leaf definition. The leaf-to-leaf distance matrix is a principal sub-matrix of the vertex-to-vertex one, and there are interlacing relations between the spectrum of a matrix and one of its principal submatrices (2). However it is not a priori the case that if two matrices have the same spectrum, then the principal submatrices with the same rows and columns will also have the same spectrum. In a similar vein, the vertex-to-vertex distance matrix can be constructed from the leaf-to-leaf distance, but the construction involves considering whether certain linear inequalities hold and so it isn't a procedure that will, a priori, transform spectra in a simple way.

More recently, Bhamidi, Evans, and Sen [24] have proven that, subject to weak general conditions, many ensembles of random trees have the property that, with probability converging to one as the number of leaves goes to infinity, a realization shares its spectrum with another tree. Their conditions are easiest to check when the ensemble can be embedded in a general continuous-time branching process where individuals give birth to a possibly random number of offspring at the arrival times of a point process up to a possibly infinite death time, and those offspring go on to behave as independent copies of their parent. This particular framework covers examples such as random recursive trees, linear preferential attachment trees, uniform rooted unordered labeled trees, and Yule trees. However, we have been unable to embed the ensemble considered here in a suitable continuous-time branching process or otherwise check the general conditions of [24].

The computer code used in this paper was written in OCaml[25] and has been made available at http://github.com/matsen/ubiquity_synonymity, along with the results produced by this code.

### Algebraic preliminaries concerning spectra and immanantal polynomials

#### Equality of adjacency and Laplacian spectra implies equality of immanantal polynomials

In order to prove results for the adjacency and Laplacian matrices simultaneously, we define for a tree *T *and arbitrary real numbers *y *and *z *the *generalized Laplacian *$\tilde{L}\left(T\right):=yD\left(T\right)+zA\left(T\right)$ (recall that *A*(*T*) is the adjacency matrix and *D*(*T*) is the diagonal matrix of vertex degrees). We define the corresponding *generalized Laplacian immanantal polynomial *of the tree *T *with *r *vertices to be

$$x\mapsto I_{\chi }\left(xI-\tilde{L}\left(T\right)\right)$$

for an irreducible character *χ *of the symmetric group *S*_*r*_.

The generalized Laplacian immanantal polynomial can be computed in a simple combinatorial fashion as follows. Define a *k-matching *to be a set of *k *pairwise disjoint edges of the tree (that is, a set of edges such that no two share a common vertex). Let *M*_*k*_(*T*) denote the set of *k*-matchings on the tree *T*. We think of an edge as a pair of vertices, so when we use the notation i∉p for a vertex *i *and a matching *p*, we mean that *i *is not one of the ends of any edge in *p. *Let *C*_*k *_denote the conjugacy class of the symmetric group *S*_*r *_consisting of permutations that are the product of *k *disjoint transpositions, and write χ(*C*_*k*_) for the common value of the character *χ *on such permutations. The following lemma appears in [14] and is included for completeness.

**Lemma 1**. *The generalized Laplacian immanantal polynomial of the tree T for the character χ is given by*

$$\underset{k\geq 0}{\sum }\chi \left(C_{k}\right)z^{2k}\underset{p\in M_{k}\left(T\right)}{\sum }\underset{i\notin p}{\prod }\left(x-yd_{i}\left(T\right)\right)$$

*where d*_*i*_(*T*) *is the degree of vertex i in the tree T*.

*Proof. *Set $M:=xI-\tilde{L}\left(T\right)=\left(m_{ij}\right)$ so that the generalized Laplacian immanantal polynomial is

(1) $$\underset{\sigma \in S_{n}}{\sum }\chi \left(\sigma \right)\underset{i}{\prod }m_{i\sigma \left(i\right)}.$$

The matrix entries *m*_*ij *_are zero unless *i *= *j *or there is an edge between *i *and *j. *If the permutation *σ *has a cycle of length 3 or greater, then corresponding term in (1) must be zero because otherwise the tree would have a loop. Therefore we need only consider permutations that are products of disjoint transpositions where, moreover, each transposition exchanges the two vertices of an edge. Such a permutation is equivalent in an obvious way to a *k*-matching for some *k*, and the lemma follows.

**Lemma 2**. *Two trees have the same spectrum for their generalized Laplacian if and only if they have the same generalized Laplacian immanantal polynomial for all characters.*

*Proof. *One direction is trivial: if two trees have the same generalized Laplacian immanantal polynomial for all characters, then their generalized Laplacians have the same characteristic polynomial and hence the same spectrum.

Conversely, if the generalized Laplacians of two trees have the same spectrum, then the characteristic polynomials of the generalized Laplacians are the same. Lemma 1 in the case *χ *= sgn, the fact that sgn(*C*_*k*_) = ±1 ≠ 0 for all *k*, and the fact that two equal polynomials have the same coefficients imply that the quantity

$$\underset{p\in M_{k}\left(T\right)}{\sum }\underset{i\notin p}{\prod }\left(x-yd_{i}\left(T\right)\right)$$

is the same for both trees for all *k. *Another application of Lemma 1 completes the proof.

#### A sufficient condition for two trees to have the same adjacency or Laplacian spectrum

We use the phylogenetic rather than graph-theoretic definition of a subtree as follows. Define the *distal vertex *of an edge to be the vertex farthest from the root that touches that edge. Then, a subtree of a given rooted tree is what results from separating an edge from its distal vertex, which then becomes the root of the subtree.

Recall that *M*_*k*_(*T*) is the set of *k*-matchings of the tree *T*. Let *N*_*k*_(*T*) be the set of *k*-matchings where the chosen edges do not contact the root.

Define

$$\begin{array}{c} P_{k}\left(T\right):=\underset{p\in M_{k}\left(T\right)}{\sum }\underset{i\notin p}{\prod }\left(x-yd_{i}\left(T\right)\right)\\ Q_{k}\left(T\right):=\underset{p\in N_{k}\left(T\right)}{\sum }\underset{i\notin p}{\prod }\left(x-yd_{i}\left(T\right)\right). \end{array}$$

The following lemma is implicit in [14], but again we include a proof for completeness.

**Lemma 3**. *Let S*_1 _*and S*_2 _*be trees with the same number of leaves. If P*_*k*_(*S*_1_) = *P*_*k*_(*S*_2_) *and Q*_*k*_(*S*_1_) = *Q*_*k*_(*S*_2_) *for all k, then any tree with S*_1 _*as a subtree has the same generalized Laplacian spectrum as the tree obtained by substituting S*_2 _*for S*_1_.

*Proof*. Let *T*_1 _be a tree with *S*_1 _as a subtree, and write *T*_2 _for the tree obtained by substituting *S*_2 _for *S*_1_. Denote by *e*_0 _the edge that connects the rest of *T*_1 _(resp. *T*_2_) to the root of *S*_1 _(resp. *S*_2_).

We differentiate between two types of *k*-matchings of *T*_*i*_: those that contain *e*_0 _and those that do not. Note that a *k*-matching of *T*_*i *_that **does not **contain *e*_0 _restricts to an ℓ-matching of *S*_*i *_for some ℓ, and all matchings of *S*_*i *_arise via such a restriction. Similarly, a *k*-matching of *T*_*i *_that does contain *e*_0 _restricts to an ℓ-matching of *S*_*i *_with the property that the root of *S*_*i *_does not belong to any edge in the matching, and all matchings of *S*_*i *_with this property arise via such a restriction.

Consider the formula for the characteristic polynomial of the generalized Laplacian matrix that comes from Lemma 1 with *χ *= sgn. Apply this formula to *T*_1 _and *T*_2_. The assumption *P*_*k*_(*S*_1_) = *P*_*k*_(*S*_2_) (resp. *Q*_*k*_(*S*_1_) = *Q*_*k*_(*S*_2_)) ensures that the matchings that do not include (resp. do include) *e*_0 _make the same contribution to the respective characteristic polynomials.

The trees depicted in Figure 1 (b) are the smallest pair of rooted bifurcating trees satisfying the criteria of this lemma. The verification of this fact was done by computer, and the corresponding *P*_*k *_and *Q*_*k *_polynomials are available in the code repository.

#### A sufficient condition for two trees to have the same distance matrix spectrum

We first recall an identity for determinants of partitioned matrices. If

$$C=\left(\begin{array}{cc} C_{11} & C_{12}\\ C_{21} & C_{22} \end{array}\right),$$

then

(2) $$\begin{array}{c} detC=\text{det}\left(\left(\begin{array}{cc} I & -C_{12}C_{22}^{-1}\\ 0 & I \end{array}\right)C\left(\begin{array}{cc} I & 0\\ -C_{22}^{-1}C_{21} & I \end{array}\right)\right)\\ =\text{det}\left(\begin{array}{cc} C_{11}-C_{12}C_{22}^{-1}C_{21} & 0\\ 0 & C_{22} \end{array}\right)\\ \;=\text{det}\left(C_{22}\right)\text{det}\left(C_{11}-C_{12}C_{22}^{-1}C_{21}\right)\\ \;=\text{det}\left(C_{11}\right)\text{det}\left(C_{22}-C_{21}C_{11}^{-1}C_{21}\right). \end{array}$$

**Lemma 4**. *Form two trees T*_1 _*and T*_2 _*by gluing the roots of trees S*_1 _*and S*_2 _*with distance matrices A*_1 _*and A*_2 _*onto the same leaf of a common tree R. Write a*_*i *_*for the column vector of distances from the leaves of S*_*i *_*to the root of S*_*i*_. *Suppose that the following pairs of matrices have the same spectra (where ' denotes transpose):*

$$\begin{array}{c} A_{i},\;i=1,2,\\ \left(\begin{array}{cc} A_{i} & a_{i}\\ {a^{\prime }}_{i} & 0 \end{array}\right),\;i=1,2,\\ \left(\begin{array}{cc} A_{i} & a_{i}\\ 1^{\prime } & 0 \end{array}\right),\;i=1,2, \end{array}$$

and

$$\left(\begin{array}{cc} A_{i} & 1\\ 1^{\prime } & 0 \end{array}\right),\;i=1,2,$$

*where ***1 ***is a column vector with each entry *1. *Then, the distance matrices of T*_1 _*and T*_2 _*have the same spectrum*.

*Proof*. Write *B *for the distance matrix of *R*. Then, *B *has the partitioned form

$$\left(\begin{array}{cc} \overset{⌣}{B} & b\\ b^{\prime } & 0 \end{array}\right),$$

where $\overset{⌣}{B}$ is the distance matrix of the tree obtained from *R *by deleting the last leaf, *b *is the column vector of distances from the other leaves of *R *to the last leaf. Assume without loss of generality that this last leaf is the attachment point of the *S*_*i*_.

Denote by *D*_*i *_the distance matrix of *T*_*i*_. Observe that

$$D_{i}=\left(\begin{array}{cc} \overset{⌣}{B} & b1^{\prime }+1a_{i}^{\prime }\\ a_{i}1^{\prime }+1b^{\prime } & A_{i} \end{array}\right).$$

Hence, by (2), *D*_*i *_has the characteristic polynomial

$$\begin{array}{llll} \text{det}\left(xI-D_{i}\right) & =\text{det}\left(xI-A_{i}\right)\text{det}\left[\left(xI-\overset{⌣}{B}\right)-\left(-b1^{\prime }-1a_{i}^{\prime }\right){\left(xI-A_{i}\right)}^{-1}\left(-a_{i}1^{\prime }-1b^{\prime }\right)\right]\; & & \;\\ & =\text{det}\left(xI-A_{i}\right)\text{det}\left[\left(xI-\overset{⌣}{B}\right)\right)\; & & \;\\ & \;\;\;\;-\left(1^{\prime }{\left(xI-A_{i}\right)}^{-1}a_{i}\right)b1^{\prime }\; & & \;\\ & \;\;\;\;-\left(1^{\prime }{\left(xI-A_{i}\right)}^{-1}1\right)bb^{\prime }\; & & \;\\ & \;\;\;\;-\left(a_{i}^{\prime }{\left(xI-A_{i}\right)}^{-1}a_{i}\right)11^{\prime }\; & & \;\\ & \left(\;\;\;\;-\left(a_{i}^{\prime }{\left(xI-A_{i}\right)}^{-1}1\right)1b^{\prime }\right].\; & & \;\\ & \; & \end{array}$$

Using (2) again, we see that a partitioned matrix of the form

$$\left(\begin{array}{cc} A & g\\ h^{\prime } & 0 \end{array}\right),$$

where *g *and *h *are column vectors, has characteristic polynomial

$$\text{det}\left(xI-A\right)\left[x-h^{\prime }{\left(xI-A\right)}^{-1}g\right],$$

and the result follows.

It was verified by computer that the trees in Figure 1 (b) are the smallest such that have distance matrices *A*_*i *_and vectors *a*_*i *_satisfying the criteria of this lemma. The corresponding characteristic polynomials are available in the code repository. We note with surprise that the smallest pair of trees with the exchange property for the distance matrix are the same as the smallest pair with the exchange property for the generalized Laplacian; this is a curiosity for which we do not have an explanation.

### Asymptotic numbers of trees

As outlined in the Introduction, the proof of Theorem 1 follows immediately from Lemma 2, Lemma 3, Lemma 4, the existence of the trees in Figure 1 (b), and the following result.

**Proposition 1**. *Let T be a rooted tree. Let t*_*n *_*be the number of trees with n leaves. Let s*_*n *_*be the number of such trees that do not contain T as a rooted subtree. Then, the fraction s*_*n*_*/t*_*n *_*goes to zero as n goes to infinity.*

*Proof. *Suppose that *T *has *a *leaves. Let $f\left(x\right):=\sum_{i=1}^{\infty }t_{i}x^{i}$ and $f_{a}\left(x\right):=\sum_{i=1}^{\infty }s_{i}x^{i}$ denote the generating functions for *t*_*n *_and *s*_*n*_, respectively. Write *ρ *for the radius of convergence of the power series *f *and *ρ*_*a *_for the radius of convergence of the power series *f*_*a*_. Note that *ρ *≤ *ρ*_*a *_< 1.

It is shown in [26] that *ρ = *0.402698 ... and

$$\underset{n\to \infty }{\text{lim}}n^{3/2}\rho^{n}t_{n}=\eta ,$$

where *η = *0.7916032 ... (see [27] for an asymptotic expansion of *t*_*n *_that extends this result and [28-31] for reviews of general methods for determining asymptotic numbers of trees of various sorts from a knowledge of the functional equations that their generating functions solve). Since *s*_*n *_is *o*(*α*^-*n*^) for any 0 <*α *<*ρ*_*a*_, it follows that *s*_*n*_*/t*_*n *_is *o*(*β*^*n*^) for any *β > ρ*/*ρ*_*a*_, and the proposition will hold if we can show that *ρ *<*ρ*_*a*_.

For the sake of completeness and because it serves as a good introduction to the derivation of the functional equation satisfied by the generating function of *s*_*n*_, we first derive the well-known functional equation satisfied by the generating function of the *t*_*n*_. See the comments after the proof of the lemma for some remarks about the history of the latter generating function.

By decomposing a tree into the two subtrees rooted at the daughters of the root, it is clear that

$$\begin{array}{cc} t_{n}=\;\;\;t_{1}t_{n-1}+t_{2}t_{n-2}+\cdots +t_{m-1}t_{m+1}, & \;\;\;\;\text{for}\;n=2m+1,\\ t_{n}=\;t_{1}t_{n-1}+t_{2}t_{n-2}+\cdots +t_{m-1}t_{m+1}+\left(\begin{array}{c} \begin{array}{c} t_{m}\\ 2 \end{array} \end{array}\right)+t_{m}, & \text{for}\;n=2m. \end{array}$$

These expressions are equivalent to the statement

(3) $$\overset{n-1}{\underset{i=1}{\sum }}t_{i}t_{n-i}=2t_{n}-t_{n/2}$$

where *t*_*n*/2 _is set to zero if *n *is odd.

From (3) the generating function *f *satisfies the functional equation

$$\begin{array}{llll} f^{2}\left(x\right) & =\overset{\infty }{\underset{n=2}{\sum }}x^{n}\overset{n-1}{\underset{i=1}{\sum }}t_{i}t_{n-i}\; & & \;\\ & =\overset{\infty }{\underset{n=2}{\sum }}x^{n}\left(2t_{n}-t_{n/2}\right)\; & & \;\\ & =2f\left(x\right)-f\left(x^{2}\right)-2x.\; & & \;\\ & \; & \end{array}$$

It will be convenient to consider the function *g*: = 1 - *f*, which satisfies the functional equation

(4) $$g\left(x^{2}\right)=2x+g^{2}\left(x\right).$$

It is shown in [15] that:

• The radius of convergence *ρ *is strictly positive.

• The functional equation (4) has a unique solution in the whole complex plane, and this solution agrees with our power series in {*x *∈ ℂ: |*x*| <*ρ*}.

• If, with a slight abuse of notation, we also denote this solution by *g*, then *g*(*ρ*) = 0.

• The point *ρ *is the only zero of *g *within {*x *∈ ℂ : |*x*| < 1}.

It is clear from the power series that *g *is continuous and decreasing on [0, *ρ*) and *g*(0) = 1. Hence *g *is strictly positive on [0, *ρ*).

As observed in [15], these observations suggest a method for computing *ρ. *Put $h\left(x\right)=g\left(x\right)/\sqrt{x}$, so that *h *satisfies *h*(*x*^2^) = 2 + *h*^2^(*x*). Set

$$w_{k}\left(\eta \right):={\left(2+\eta^{2^{k}}\right)}^{2^{-k}},\;\eta \in \mathbb{R},$$

and

$$q_{n}:=w_{n-1}\circ w_{n-2}\cdots \circ w_{0},$$

so that each function *q*_*n *_is strictly increasing on [-2, +∞) and *q*_1 _≤ *q*_2 _≤ .... In particular, lim_*n*→∞ _*q*_*n*_(*y*) exists in ℝ ∪ {+∞} for each *y *∈ [-2, +∞). Moreover,

$$\underset{n\to \infty }{\text{lim}}q_{n}\left(h^{2}\left(x\right)\right)=\underset{n\to \infty }{\text{lim}}{\left(h\left(x^{2^{n}}\right)\right)}^{2^{1-n}}=\underset{n\to \infty }{\text{lim}}\frac{{\left(g\left(x^{2^{n}}\right)\right)}^{2^{1-n}}}{x}=\frac{1}{x}$$

for 0 <*x *< 1. Therefore

$$\frac{1}{\rho }=\underset{n\to \infty }{\text{lim}}q_{n}\left(0\right).$$

Conveniently, (3) holds with *s*_*n *_in place of *t*_*n *_for all *n *except for *n *= *a*; in this case one simply adds two to the right hand side of the equation to make up for the fact that *s*_*a *_= *t*_*a *_- 1. Hence *f*_*a *_satisfies the functional equation.

(5) $$f_{a}^{2}\left(x\right)=2f_{a}\left(x\right)-f_{a}\left(x^{2}\right)-2x+2x^{a}.$$

Set *g*_*a*_: = 1 - *f*_*a*_, so that

(6) $$g_{a}\left(x^{2}\right)=2x-2x^{a}+g_{a}^{2}\left(x\right).$$

It is clear that *g*_*a *_is continuous and decreasing on [0, *ρ*_*a*_) and *g*_*a*_(0) *= *1. Following the arguments in [15], the functional equation (6) has a unique solution in the whole complex plane, and this solution agrees with our power series in {*x *∈ ℂ: |*x*| <*ρ*_*a*_}. Moreover, analogues of the other properties of *g *obtained in [15] hold for *g*_*a*_.

Set $h_{a}\left(x\right)=g_{a}\left(x\right)/\sqrt{x}$, so that

$$h_{a}\left(x^{2}\right)=2-2x^{a-1}+h_{a}^{2}\left(x\right).$$

Put

$$w_{k,a,\xi }\left(\eta \right):={\left(2-2\xi^{2^{k}}+\eta^{2^{k}}\right)}^{2^{-k}},\;\eta \in \mathbb{R},$$

and

$$q_{n,a,\xi }:=w_{n-1,a,\xi }\circ w_{n-2,a,\xi }\cdots \circ w_{0,a,\xi }.$$

Then

$$\underset{n\to \infty }{\text{lim}}q_{n,a,x^{a-1}}\left(h_{a}^{2}\left(x\right)\right)=\underset{n\to \infty }{\text{lim}}{\left(h_{a}\left(x^{2^{n}}\right)\right)}^{2^{1-n}}=\underset{n\to \infty }{\text{lim}}\frac{{\left(g_{a}\left(x^{2^{n}}\right)\right)}^{2^{1-n}}}{x}=\frac{1}{x}$$

for 0 <*x *< 1, and, in particular,

$$\frac{1}{\rho_{a}}=\underset{n\to \infty }{\text{lim}}q_{n,a,\rho_{a}^{a-1}}\left(0\right).$$

Now

$$\begin{array}{llll} q_{n,a,\rho_{a}^{a-1}}\left(0\right) & =w_{n-1,a,\rho_{a}^{a-1}}\circ w_{n-2,a,\rho_{a}^{a-1}}\cdots \circ w_{0,a,\rho_{a}^{a-1}}\left(0\right)\; & & \;\\ & \leq w_{n-1}\circ w_{n-2}\cdots \circ w_{1}\circ w_{0,a,\rho_{a}^{a-1}}\left(0\right)\; & & \;\\ & =q_{n}\left(-2\rho_{a}^{a-1}\right),\; & & \;\\ & \; & \end{array}$$

and so

$$\frac{1}{\rho_{a}}\leq \underset{n\to \infty }{\text{lim}}q_{n}\left(-2\rho_{a}^{a-1}\right)\leq \underset{n\to \infty }{\text{lim}}q_{n}\left(0\right)=\frac{1}{\rho }.$$

It therefore suffices to show that the function *y *↦ lim_*n*→∞ _*q*_*n*_(*y*) is strictly increasing on (-*ε*, +∞) for some 0 <*ε *< 2.

Observe that the derivative of *q*_*n *_satisfies

$$q_{n}^{\prime }=\overset{n-1}{\underset{k=1}{\prod }}w_{k}^{\prime }\circ q_{k}.$$

For *k *≥ 1,

$$\begin{array}{llll} w_{k}^{\prime }\left(x\right) & =x^{2^{k}-1}{\left(2+x^{2^{k}}\right)}^{2^{-k}-1}\; & & \;\\ & ={\left(2x^{-2^{k}}+1\right)}^{2^{-k}-1},\; & & \;\\ & \; & \end{array}$$

so that $x\mapsto w_{k}^{\prime }\left(x\right)$ is non-decreasing for *x *> 0. For *y *∈ (-*ε*, +∞),

$$w_{k}^{\prime }\circ q_{k}\left(y\right)\geq w_{k}^{\prime }\circ q_{1}\left(y\right)\geq w_{k}^{\prime }\circ q_{1}\left(-\varepsilon \right)=w_{k}^{\prime }\left(2-\varepsilon \right)$$

and hence

$$\underset{n\to \infty }{\text{lim }\text{inf}}\underset{y>-\varepsilon }{\text{inf}}q_{n}^{\prime }\left(y\right)\geq \overset{\infty }{\underset{k=1}{\prod }}w_{k}^{\prime }\left(2-\varepsilon \right).$$

Taking 0 <*ε *< 1, the proof will be completed by demonstrating for any *x *> 1 that

$$\overset{\infty }{\underset{k=1}{\prod }}w_{k}^{\prime }\left(x\right)>0.$$

Taking the logarithm gives

$$\begin{array}{llll} \overset{{}_{\infty }}{\underset{k=1}{\sum }}\left(2^{-k}-1\right)\text{log}\left(2x^{-2^{k}}+1\right) & >-\overset{\infty }{\underset{k=1}{\sum }}\text{log}\left(2x^{-2^{k}}+1\right),\; & & \;\\ & >-\overset{\infty }{\underset{k=1}{\sum }}2x^{-2^{k}},\; & & \;\\ & \; & \end{array}$$

and this series clearly converges by the ratio test.

In relation to Proposition 1, we note from [13] that the number of rooted strictly bifurcating trees without a given subtree is asymptotically smaller than the number of all graph-theoretic trees (see also [32] for more about the enumeration of general trees without a given subtree), but this is not enough for our purposes. We needed to show that it is asymptotically smaller than the space of all rooted strictly bifurcating trees. The generating function for *t*_*n *_seems first to have been investigated in [15] in connection with enumerating "types of arrangements" in a commutative but non-associative algebra, such as *a*_1_(*a*_2_(*a*_3_*a*_4_)) or (*a*_1_*a*_2_)(*a*_3_*a*_4_); these are identical to rooted bifurcating trees in the "Newick" format [1]. The recursion behind the generating function has been re-discovered independently several times such as in [33] - see [34] for a discussion and several further references. We remark that numerically iterating the quantity *q*_*n *_of the proof converges quickly to the value of *ρ*^-1 ^calculable by other means [26,35]. We also observe that the methods of [27-31] can be used to show, in the notation of the proof, that $\underset{n\to \infty }{\text{lim}}n^{3/2}\rho_{a}^{n}s_{n}=\eta_{a}$ for some positive constant *η*_*a *_and hence lim_*n*→∞_(*ρ*_*a*_/*ρ*)^*n*^(*s*_*n*_/*t*_*n*_) = *η*_*a*_/*η*, but we don't pursue this matter here.

### Numerical experiments

Proposition 1 says nothing about the rate of convergence of the fraction. Here we investigate this rate using computation. The characteristic polynomials for the generalized Laplacian were calculated via a doubly-recursive algorithm to enumerate matchings. The characteristic polynomials for the distance matrices were calculated via the Leverrier-Faddeev algorithm [36].

**Figure 2:**
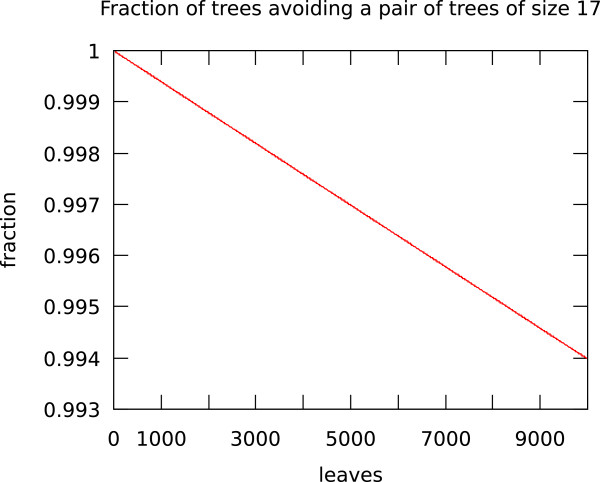
**The fraction of trees not containing a given pair of subtrees of size 17 **.

Table 1 shows that the fraction of trees with unique spectra does not go to zero very quickly. We can't compute this fraction for large numbers of leaves, but we can get some idea of the convergence by using the recursion relation corresponding to the generating function (5). Figure 2 shows the number of trees that do not have one of two subtrees of size seventeen as a subtree. This is an actual fraction that can be used with Proposition 1 in order to prove Theorem 1 for the generalized Laplacian matrix.

**Table 1:** The number of trees, the number of spectra for the generalized Laplacian (GLS), and the number of spectra for the distance matrix (DS).

leaves	trees	GLS	DS
2	1	1	1
3	1	1	1
4	2	2	2
5	3	3	3
6	6	6	6
7	11	11	11
8	23	22	23
9	46	45	46
10	98	95	98
11	207	203	207
12	451	443	451
13	983	972	983
14	2179	2159	2179
15	4850	4827	4850
16	10905	10870	10905
17	24631	24580	24630
18	56011	55931	56009
19	127912	127830	127908

Figure 2 shows that this fraction converges extremely slowly, despite the fact that as shown above it is asymptotically equivalent to *β*^*n *^for some 0 <*β *< 1. It is important to note, however, that this fraction is probably a very crude upper bound on the fraction of trees that share a spectrum with another tree. As can be seen in Table 1, the actual number not sharing a spectrum goes down considerably more quickly, though it is probably still the case that the vast majority of trees of intermediate size should have their own spectra.

## Conclusions

Spectral invariants of matrix formulations of trees are a natural way to quantify the shape of phylogenetic trees. However, in this paper we show that a complete classification of tree shapes using common spectral invariants of generalized Laplacian and distance matrices is not possible. For either of these choices of matrix we show that the fraction of binary trees with a unique spectrum goes to zero as the number of leaves goes to infinity, but the rate of convergence of the above fraction to zero appears to be slow. For the adjacency and Laplacian matrices, we show that the *a priori *more informative immanantal polynomials have no greater power to distinguish between trees.

## Competing interests

The authors declare that they have no competing interests.

## Authors' contributions

FAM conceived of the project, proved the theorems, performed the numerical experiments, and wrote the paper. SNE applied the immanant, proved the theorems, and wrote the paper.
